# From Bedside to Diagnosis: The Role of Ocular Fundus in Systemic Infections

**DOI:** 10.3390/jcm12237216

**Published:** 2023-11-21

**Authors:** Giacomo Visioli, Marco Zeppieri, Valeria Iannucci, Priscilla Manni, Giuseppe Maria Albanese, Carlo Salati, Leopoldo Spadea, Maria Pia Pirraglia

**Affiliations:** 1 Department of Sense Organs, Sapienza University of Rome, Viale del Policlinico 155, 00161 Rome, Italy; giacomo.visioli@uniroma1.it (G.V.);; 2 Department of Ophthalmology, University Hospital of Udine, 33100 Udine, Italy; 3 Eye Clinic, Policlinico Umberto I University Hospital, 00161 Rome, Italy

**Keywords:** systemic infections, bedside diagnosis, ocular fundus examination, clinical practice, ophthalmology consultation, infection management

## Abstract

In this comprehensive review, we delve into the significance of the ocular fundus examination in diagnosing and managing systemic infections at the bedside. While the utilization of advanced ophthalmological diagnostic technologies can present challenges in bedside care, especially for hospitalized patients confined to their beds or during infection outbreaks, the ocular fundus examination often emerges as an essential, and sometimes the only practical, diagnostic tool. Recent discussions have highlighted that the role of an ocular fundus examination might not always be advocated as a routine diagnostic procedure. With this context, we introduce a decision tree tailored for assessing the ocular fundus in inpatients with systemic infections. We also present an overview of systemic infections that impact the eye and elucidate key signs detectable through a bedside ocular fundus examination. Targeted primarily at non-ophthalmology clinicians, this review seeks to offer a comprehensive insight into a multifaceted approach and the enhancement of patient clinical outcomes.

## 1. Introduction

Ocular fundus examination offers a privileged window, providing a unique, non-invasive means of observing vasculature in vivo and subsequently uncovering numerous systemic diseases. Even though the ocular fundus is generally considered an invaluable diagnostic tool, its application can be potentially underappreciated in the context of bedside examinations for patients admitted with various systemic conditions [1]. The challenges incumbent upon the employment of the new advanced ophthalmic diagnostic technologies, particularly in bedside environments and amidst infection outbreaks, are multifaceted [2,3,4]. Impediments include logistical issues related to instrument transport to bedside settings and the potential for enhanced risk of cross-infection when deploying such instrumentation in infectious contexts [5,6,7]. Thus, the binocular indirect ophthalmoscope (BIO) remains an expedient, cost-effective diagnostic tool capable of rapid deployment in varied settings, including in situ patient care environments [8].

Numerous systemic infectious diseases can present diagnostic indicators within the ocular fundus, manifesting in both life-threatening conditions such as septicemia or endocarditis, and in infections from specific agents like *Cytomegalovirus* (CMV), human immunodeficiency virus (HIV), tuberculosis (TB), syphilis, and toxoplasmosis [9,10,11]. These agents often bear distinct signatures within the ocular fundus that may be detected during a comprehensive examination. Notably, these anomalies may present even in the absence of overt ophthalmic symptoms, meaning the ophthalmologist could, in certain instances, be pivotal in rendering initial diagnoses of these systemic conditions based on ocular findings [12].

This review aims to underscore the role of ocular fundus examination in the context of systemic infections. As recent guidelines have shifted away from routine ophthalmoscopy, determining when such examinations are pivotal will be the focus of our discussion. Secondly, we provide a detailed decision tree to facilitate the streamlined application of ocular fundus examination in the identification and management of ocular infections resulting from systemic disease. Lastly, the implications of the findings on patient management will be discussed, emphasizing how outcomes may vary based on examination results. Through a review of existing literature and present clinical practices, this review endeavors to amplify the cognizance and practical application of ocular fundus examinations among clinicians, thereby fortifying its role in diagnostic protocols related to systemic infections.

## 2. Methods

A literature search was conducted across several research databases, including PubMed, Scopus, and Web of Science, limiting articles to those published within the past 30 years. Keywords used to retrieve relevant documents included “ocular fundus examination”, “systemic infections”, “bedside diagnosis”, “infectious diseases and eye”, “ocular manifestations”, “retinal findings”, “binocular indirect ophthalmoscopy”, and “endogenous infections and eye”. Boolean operators (AND, OR) were employed to refine the search and navigate terminology variations across different databases. Studies discussing ocular fundus examination and its utility in detecting systemic infections, ophthalmic manifestations of such infections, and its application in bedside diagnostics were included. When categorizing the onset of ocular findings, we classified them as ‘early’ or ‘late’ based on their presentation, with ‘early’ referring to symptoms more likely appearing days to weeks after infection and ‘late’ to those manifesting months to years post-infection [13]. These categorizations would only serve as a general guide, since the actual timing is subject to a multitude of variables, including the patient’s clinical conditions.

## 3. Ocular Fundus Examination

**Figure 1:**
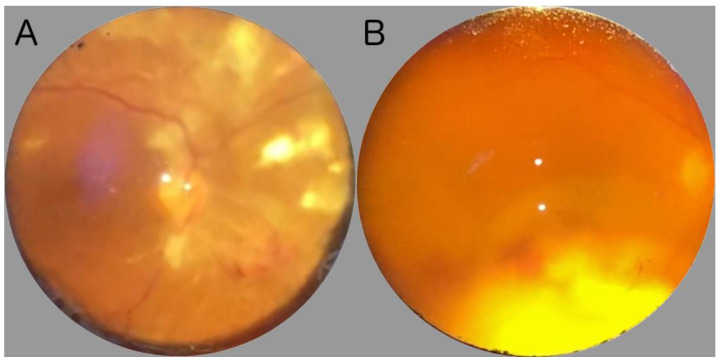
Examples of two real-world fundus examinations conducted in infectious disease wards and photographed using a smartphone camera. (**A**) shows a severe case of necrotizing herpetic retinopathy at the posterior pole. (**B**) despite vitreous opacity, a wide area of retinal exudate with hemorrhage at the edges near the optic nerve can be recognized. The patient was ultimately diagnosed with systemic nocardiosis.

Ocular fundus examination allows for the inspection of the posterior segment of the eye, including the vitreous, retina, optic nerve, macula, and retinal vessels [14]. Ocular fundus can be explored by direct or indirect ophthalmoscopy [15]. Direct ophthalmoscopy procures an upright, unreversed image with a magnification of around 15 times, whereas indirect ophthalmoscopy delivers a reversed, inverted image, magnified between two to five times. The latter, which has a longer learning curve, can be subdivided into monocular indirect ophthalmoscopy (MIO) and binocular indirect ophthalmoscopy (BIO). Direct ophthalmoscopy is suitable for swift assessments of the optic nerve head or evaluating the red reflex, and is frequently employed by non-ophthalmologist clinicians, such as neurologists and pediatricians [16,17]. Conversely, indirect ophthalmoscopy, particularly the BIO, provides a stereoscopic, extensive view of the retina, encompassing around a 40–45-degree field when utilizing a 20D lens, enabling a more detailed examination and evaluation of peripheral retinal structures, and allowing dynamic observation through lens movement and scleral depression [18]. Furthermore, during ophthalmoscopy, the anterior segment of the eye can also be evaluated at the bedside. Notably, signs of anterior segment involvement, such as corneal ulcers or abscesses, or the presence of synechiae can be also detected, even if in a ward context [7]. In Figure 1, two examples from real-world fundus examinations conducted in infectious disease wards using a 20D lens are shown.

Instances warranting an ophthalmologist’s expertise for fundus examination might include, but are not limited to, suspected retinal detachment in the context of sudden onset floaters or flashes, evaluating for hypertensive or diabetic retinopathy in patients with uncontrolled glycemia, evaluating ocular manifestations in auto-immune diseases, examining for papilledema in scenarios of suspected elevated intracranial pressure or—as described in this paper—investigating potential ocular manifestations of systemic infections [1,19]. In the subsequent section, an overview of the principal ocular fundus findings in systemic infections, categorized according to etiological agents—bacterial, viral, fungal, and parasitic—will be described.

## 4. Principal Systemic Infections at Bedside

A considerable number of patients are admitted to hospitals with symptoms suggestive of infectious diseases, yet without an immediate, clear diagnosis. Pending results from various diagnostic procedures, such as blood tests or imaging, medical practitioners often face a diagnostic delay that could hinder timely therapeutic intervention. In this interval, the evaluation of the ocular fundus may provide a rapid and informative diagnostic tool, potentially identifying indicative signs of systemic infection and enabling the preliminary identification of an etiological agent [20]. This timely detection allows for the earlier initiation of appropriate treatments, even before exhaustive diagnostic test results are available [21]. The ensuing section will explore key ocular fundus manifestations associated with primary systemic infections, underscoring their diagnostic significance when assessed at the bedside in a clinical setting. Not encompassing all ocular findings related to systemic infections, we will specifically highlight manifestations evident in the ocular fundus. The content has been structured to provide clinicians with a clear and brief overview of these manifestations, serving as a convenient reference for diagnostic evaluations.

### 4.1. Systemic Bacterial Infections

Within the scope of systemic bacterial infections, ocular fundus changes are infrequent, primarily due to the protection provided by the blood-retina barrier (BRB) [22,23]. However, ocular involvement should be considered in cases of severe systemic bacterial infections, especially among immunocompromised patients [24,25,26]. If an infection is promptly identified within the eye, clinicians may consider changing treatment strategies such as selecting an antibiotic capable of penetrating the BRB, particularly while awaiting confirmatory blood culture results [27,28].

In certain scenarios, the identification of systemic infections, such as endocarditis or TB, might initially surface during an ophthalmologic examination [29]. For example, in cases where endocarditis does not present clear valvular vegetations upon transthoracic echocardiography, ocular signs, such as Roth spots (retinal hemorrhages with a central white or pale center), could serve as initial diagnostic clues, encouraging further investigations such as transesophageal echocardiogram [30,31]. Similarly, in TB, choroiditis might provide early indications of the disease, especially when other systemic signs are subtle or gradually progressive. In Table 1 the principal bacterial infections along with the most common fundus findings are presented [32,33,34,35,36,37,38,39,40].

### 4.2. Systemic Viral Infections

Ocular fundus examination could uncover underlying systemic viral infections and potentially severe health conditions, providing critical insights particularly relevant in immunocompromised populations [41,42]. In naïve HIV patients, retinal microangiopathy is commonly observed. It frequently presents as cotton wool patches, which are pale, fluffy lesions on the retina, caused by microinfarctions of the retinal nerve fiber layer due to obstructed retinal capillaries [43,44]. This manifestation is especially prevalent with decreased CD4 lymphocyte counts, serving as a potential marker for monitoring disease progression and severity. Additionally, viral infections such as CMV retinitis, present with distinctive retinal findings, including vascular-distributed, hemorrhagic, or granular retinitis, providing a diagnostic marker, especially crucial in contexts of immunosuppression, whether due to HIV or other etiologies like organ transplantation [45,46]. Detection of CMV infection in the eye, beyond its ocular implications, signals a vital alert for possible life-threatening systemic involvement, thereby underscoring the importance of ocular assessments in comprehensive patient management, especially amidst immunosuppressive conditions [47,48]. In Table 2, principal viruses, along with their ocular fundus findings, are reported [49,50,51,52,53,54].

Herpes viruses can cause serious necrotizing conditions that affect the retina. In this context, necrotizing herpetic retinopathy (NHR) is a collective term for a group of diseases that cause acute retinal necrosis due to herpes viruses, encompassing conditions such as CMV retinitis, acute retinal necrosis (ARN), and progressive outer retinal necrosis (PORN) [55]. CMV retinitis typically occurs in immunocompromised individuals, such as those with AIDS or undergoing immunosuppressive therapy, and presents with a distinctive appearance often described as resembling “cottage cheese with ketchup”, primarily affecting the posterior pole. Vitritis is generally absent, but signs of periphlebitis may be present [56,57]. The primary treatment for CMV retinitis is antiviral therapy, commonly with valganciclovir. In contrast, ARN can affect both immunocompetent and immunocompromised individuals and usually begins in the peripheral retina. It can present with mild hemorrhages and is often accompanied by severe vitritis. ARN typically requires systemic antiviral treatment, such as aciclovir. It is often diagnosed in an outpatient setting, as inpatients may not exhibit systemic symptoms indicative of this condition. PORN, primarily seen in severely immunocompromised patients, particularly those with advanced AIDS, is characterized by rapid progression and extensive necrosis of the outer retina, often starting at the posterior pole. Unlike CMV retinitis, PORN does not typically present with significant intraocular inflammation, which is reflected in the minimal anterior chamber reaction and vitreous cell presence. The most common causative agent is the Varicella Zoster Virus (VZV), followed by HSV. Treatment for PORN includes aggressive antiviral therapy administered both intravitreally and intravenously, along with the management of disease sequelae, such as retinal detachment. Despite therapy, the visual prognosis for PORN remains poor [41,51,55].

A particular discourse warrants allocation to emerging viral diseases such as SARS-CoV-2, given the proliferation of case reports delineating varied retinal findings in the context of COVID-19 [58]. Observations have highlighted various retinal changes, including cotton wool spots, inner retinal optical coherence tomography (OCT) hyperreflective spots, and retinal microhemorrhages, suggesting the systemic impact of SARS-CoV-2 beyond respiratory complications. The occurrence of vascular occlusions such as Central Retinal Vein Occlusion (CRVO) and Central Retinal Artery Occlusion (CRAO), and conditions like Acute Macular Neuroretinopathy (AMN) and Paracentral Acute Middle Maculopathy (PAMM) further emphasize the potential ocular involvement in infected patients. As research progresses, it is crucial to determine whether these retinal manifestations are directly attributable to the virus or are influenced by other concurrent factors. In fact, the ambiguity persists due to a pivotal consideration, the potential influence of non-COVID-19-related systemic afflictions, such as hypertensive or diabetic retinopathy, on these retinal findings cannot be sidelined [59]. Indeed, our prior research, which scrutinized retinal conditions in a cohort of 43 inpatients with severe COVID-19, did not affirmatively identify specific retinal anomalies attributable to the viral infection [7]. After our publication, while numerous studies have presented various retinal findings, none have decisively contravened our study, thereby not substantiating the presence of specific clinical pictures of COVID-19-related retinal pathologies. Hence, the precise relationship between SARS-CoV-2 and retinal findings remains to be further clarified [60].

### 4.3. Systemic Fungal Infections

In the context of systemic fungal infections, which predominantly affect immunocompromised individuals, conducting an ocular fundus examination is generally recommended, particularly in high-risk patients such as those who are immunocompromised, receiving intensive care, or with long-term catheterization [61,62]. Ocular manifestations often start with retinitis, then extend to the vitreous and ultimately evolve into endophthalmitis. Hence, early detection of initial ocular involvement can deter the progression to a more severe condition. Notably, fungi like *Candida* species, including *Candida albicans*, and molds such as *Aspergillus* and *Fusarium* are primary causative agents [63,64]. The initial site of involvement is often the choroid because of its extensive blood supply. The infection typically progresses from the choroid to the retina, potentially starting as choroiditis or chorioretinitis before developing into a more severe vitreal infection [65].

When a systemic fungal infection is identified, clinicians may initiate an empirical fungal treatment (e.g., Caspofungin) that strategically considers drug toxicity and pharmacokinetic properties [66,67]. However, ensuring optimal therapeutic outcomes has frequently involved a comprehensive assessment of the ocular fundus, especially since identifying ocular candidiasis dictates a pivot towards utilizing agents, such as Fluconazole, that traverse the BRB effectively [68,69]. In clinical practice, unaddressed ocular candidiasis is considered a credible threat to patient survival by maintaining a continual source of infection [70]. These common practices are more recently confirmed by recent guidelines about performing ocular fundus evaluations. In 2022, the practice of routine screening for intraocular infection stemming from *Candida* septicemia was evaluated by the American Academy of Ophthalmology (AAO), concluding that routine ophthalmologic consultations after diagnosing systemic *Candida* septicemia might be of limited value. Nevertheless, seeking ophthalmologic advice was stated as prudent for patients exhibiting signs or symptoms of an ocular infection, irrespective of a *Candida* septicemia diagnosis [63]. More insights and detailed analysis regarding recent guidelines for performing ophthalmoscopy will be discussed in the subsequent section.

### 4.4. Systemic Parasitic Infections

Parasitic incursions into the ocular milieu may emanate from a diverse array of organisms, including protozoa, nematodes, and cestodes, each engendering distinct pathological sequela within the ocular fundus [71]. Such pathologies, whether directly attributed to parasitic activity or indirectly mediated through host immune responses, pervade both the anterior and posterior ocular segments. The latter, which encompasses choroiditis, retinichoroiditis, retinal vasculitis, and additional deleterious conditions, warrants rigorous investigation to safeguard against irreversible retinal damage and concomitant visual impairment [72].

Ocular toxoplasmosis, commonly resulting from *T. gondii*, typically presents notable retinal findings such as a distinctive white focal retinitis with concurrent vitreous inflammation, often described as a “headlight in the fog” [73,74]. Alternatively, ocular toxocariasis, often stemming from *Toxocara* infestation, might display as granulomatous posterior uveitis, peripheral inflammatory masses, or even, in severe cases, retinal detachment [75]. In Table 3, the main fundus findings in systemic fungal and parasitic infections are displayed [76,77,78,79,80,81].

## 5. When Assessing Ocular Fundus in Systemic Infections

Not every patient presenting with an infection necessitates a fundus examination. Current guidelines, reflecting advances in understanding and methodologies, do not endorse routine ophthalmologic consultation for a broad spectrum of systemic infections [63,82,83]. Nevertheless, discerning when an ocular fundus examination should be pursued remains paramount.

### 5.1. The Evidence about Ophthalmoscopy in Systemic Fungal Infections

Generally, ophthalmologists have been routinely consulted in hospitals to screen for intraocular infections in patients with *Candida* bloodstream infections. This approach originated before the advent of systemic antifungal medications and before the establishment of clear definitions of ocular disease associated with candidemia. The Infectious Diseases Society of America (IDSA) and the European Society of Clinical Microbiology and Infectious Diseases (ESCMID) have provided insights into the role of fundus examinations in the context of candidemia [84,85]. The IDSA, in 2016, specifically recommends fundoscopy screening within the first week for all patients who test positive for fungal blood cultures, highlighting the potential ocular complications that can arise from candidemia. This proactive stance is driven by the fact that many patients with candidemia can be asymptomatic or may be too systemically unwell to report visual disturbances [84,85]. In stark contrast, ESCMID’s guidelines on the Diagnosis and Management of Candida Diseases make no explicit mention of ocular involvement, indicating a more conservative stance. The Royal College of Ophthalmologists (RCOphth) has also entered the discussion, collaborating with the Intensive Care Society to recommend fundoscopy screenings for Intensive Care Unit (ICU) patients with positive fungal cultures, emphasizing that such patients are more likely to be non-verbal and, therefore, less likely to communicate visual symptoms [86]. A 2018 study by El-Abiary et al. [61] conducted over two years, examined 168 adults with *Candida*-positive blood cultures. While 95.8% had *Candida* species detected, only one individual showed signs of *Candida* chorioretinitis. Given these findings, the study concluded that routine fundoscopy might not be necessary for every culture-positive patient [61]. Nevertheless, it should be noted that in this study most of the patients (48.8%) were treated with Fluconazole, which has good ocular penetration, and this could partially explain the low incidence of *Candida* chorioretinitis [87,88]. However, when alternative antifungal therapies, such as Amphotericin B or Caspofungin, are preferred—both of which have comparatively poorer penetration profiles—the strategy for ocular fundus examination might need reevaluation [88]. More recent recommendations by the AAO underscore the importance of evidence-based practices in patient care, especially concerning screening for endogenous *Candida* endophthalmitis. The institution of such guidelines aimed at eliminating low-value care practices, which not only prove inefficient but may also pose risks to patient safety. The Academy’s position on routine screening for intraocular infections resulting from *Candida* bloodstream infections seeks to minimize unnecessary examinations and aligns with the evidence presented in various studies on endogenous *Candida* endophthalmitis [63]. A systematic review in 2019 highlighted a less than 1% prevalence for endophthalmitis resulting from *Candida* septicemia in routinely screened patients [89]. Other research pointed to higher rates but had methodological limitations, including inaccuracies in ocular disease classification, absence of vitreous biopsies, selection biases, and a lack of data on longer-term visual outcomes. Moreover, some investigations attributed ocular symptoms to *Candida* infections when other comorbidities might have been responsible [90]. In the absence of definitive evidence, suggesting alterations in medical management treatment, due to ocular involvement, should be primarily guided by the systemic *Candida* infection rather than ocular manifestations. However, individualized assessments are crucial, hence, we have devised a decision tree, which will be elaborated upon in the subsequent section.

### 5.2. A Decision Tree for Clinicians

**Figure 2:**
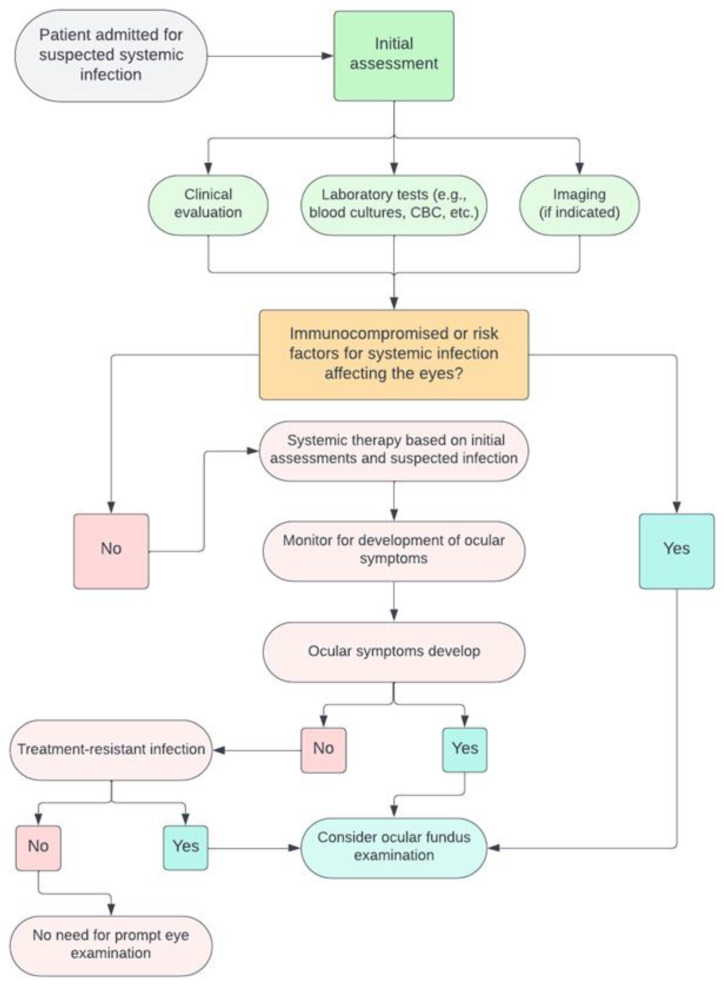
The flowchart illustrates the clinical procedure for assessing and treating patients admitted for suspected systemic infections, indicating when an ocular fundus examination is advisable.

While the significance of routine ophthalmoscopy remains a topic of discussion, various guidelines emphasize the necessity of a personalized approach. This approach should account for the distinct clinical conditions of each patient, while also staying aligned with the latest scientific consensus. Consequently, we have formulated a comprehensive set of criteria to delineate the specific scenarios where ocular fundus examination could impact patient outcomes. To begin, even if ocular symptoms are not readily apparent, practitioners should maintain a heightened level of vigilance for patients with systemic infections known for ocular involvement or possessing a notable propensity for dissemination, such as toxoplasmosis or CMV [52,77]. Firstly, even in the absence of ocular symptoms, a high index of suspicion should be reserved for patients where the systemic infection is notorious for ocular involvement or has a propensity to disseminate, e.g., toxoplasmosis or CMV [91]. It is advisable to categorize patients into risk strata, considering factors such as immunosuppression, prolonged hospitalization, or the presence of a central venous catheter which are commonly associated with systemic fungal infections. Secondly, the temporality and nature of ocular symptoms, within the framework of systemic infection, must be judiciously assessed. A patient with chronic, indolent visual blurriness spanning months might not necessitate an urgent fundus examination, as opposed to one presenting with acute visual disturbances concomitant with systemic infectious symptoms. Manifestations such as sudden vision of visual field loss, eye pain, floaters, photophobia, and altered pupil reaction might warrant an ophthalmoscopy exam. However, in the context of intensive care units (ICU), it is crucial to recognize that many patients, due to their critical state or sedation, may be unable to communicate or articulate any visual disturbances or ocular discomfort [86]. Consequently, in such settings, a proactive approach, including routine ocular evaluations or heightened vigilance for subtle clinical signs of eye involvement, becomes indispensable to ensure timely diagnosis and intervention [86]. Moreover, any systemic infection with an unidentified etiological agent, which is refractory to the current antimicrobial therapy, especially in a context where ocular symptoms are present, should trigger a fundus examination. Finally, a patient’s geographical and socio-economic context should be considered, understanding that certain parasitic infections might be more prevalent in specific locales or conditions, hence augmenting the pre-test probability in symptomatic individuals [92,93]. In Figure 2, we propose a decision tree for assessing the ocular fundus in inpatients with systemic infections. This framework is based on current guidelines and is further informed by our practical experience in performing ocular fundus examinations within infectious disease wards.

To date, comprehensive data on the implications of ocular fundus examinations for inpatients with systemic infections remain sparse, especially in terms of understanding its diagnostic efficacy, impact on treatment modifications, and ultimate contribution to patient outcomes in a hospital setting. Further retrospective studies examining historical patient data could shed light on the clinical trajectories of inpatients undergoing fundus examination versus those who were not during systemic infections. Specifically, such studies might elucidate differences in morbidity, intervention timeliness, and overall patient survival. Although prospective studies could solidify the present evidence, initiating such research could pose several ethical issues, particularly when it involves potentially withholding necessary ophthalmological consultations. Instead, more nuanced research designs, perhaps observational in nature, could be pursued. Nevertheless, the decision tree presented here can be effectively employed in developing countries, with appropriate adaptations, where the incidence of infectious diseases is high. Ocular fundus examination is an affordable and low-resource diagnostic tool that could be especially beneficial in areas where diseases such as HIV are prevalent [94]. Requiring minimal equipment, it is accessible for bedside use even in settings with limited healthcare infrastructure. Training healthcare workers in these regions to conduct and interpret ocular fundus examinations could aid in the timely detection and treatment of systemic conditions, potentially even more so than in countries where more costly diagnostic examinations are readily available.

## 6. Patient Management after Ocular Fundus Examination

Depending on the findings of the examination, clinical strategies will differ depending on conclusive and inconclusive findings. In scenarios with inconclusive evidence, monitoring ocular symptoms could be useful to understand whether ocular fundus could be re-evaluated based on the patient’s progress and the efficacy of systemic interventions. On the other hand, in cases with conclusive evidence pointing towards specific pathologies, the therapeutic strategy might necessitate modifications. This could involve enhancing the systemic antimicrobial regimen or incorporating specific antiviral/fungal treatments. Moreover, active collaboration with ophthalmology specialists becomes paramount to crafting a comprehensive care pathway.

**Figure 3:**
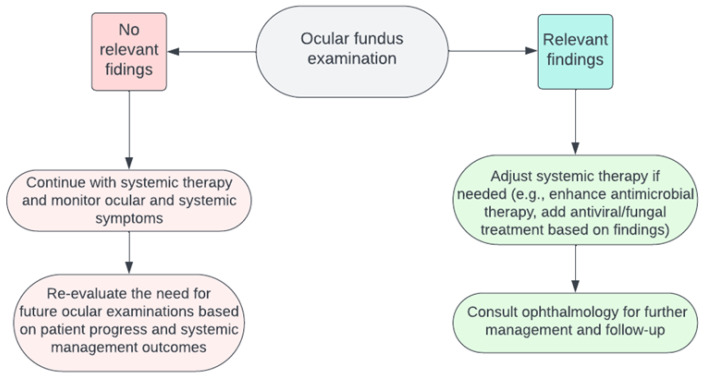
The flowchart visualizes the decision-making process following an ocular fundus examination in patients with systemic infections.

The utility of ocular fundus examination in guiding patient management is a nuanced topic. In the context of bacterial infections, detecting ocular involvement might suggest a systemic dissemination of the infection. Yet, conventional treatments for bacterial infections typically involve broad-spectrum antibiotics. These medications would inherently address bacterial pathogens impacting the eye. Therefore, the presence of ocular involvement might not substantially alter the foundational approach to treating bacterial infections. However, if the systemic infection is controlled, prompt treatment of the eye is crucial. Early intervention can help prevent potential vision loss, underscoring the significance of considering targeted eye therapies in such cases, such as performing aqueous/vitreous taps for antibiograms or contemplating intravitreal antibiotic injections [9]. Concerning some viral infections such as CMV, ocular changes can be instrumental in dictating treatment modalities. Detecting CMV retinitis, particularly in immunocompromised individuals, could necessitate the introduction or adjustment of specific antiviral agents. This finding may also underscore the importance of evaluating and addressing the patient’s overall immunological status [47]. A flowchart about patient management after ocular fundus examination is shown in Figure 3, and it emphasizes an interdisciplinary methodology that integrates diagnostic insights with the broader clinical picture to optimize patient outcomes.

## 7. Conclusions

Examining the ocular fundus demonstrates significant clinical relevance in systemic infections among inpatients. The necessity of such evaluations is not universal for all infectious cases but critical when systemic infections, notably those demonstrating a potential for ocular involvement. The utilization of a collaborative decision tree is proposed to guide clinicians in identifying patients who may derive substantial benefit from fundus examinations, thereby enhancing diagnostic accuracy and tailoring therapeutic interventions. A well-structured, interdisciplinary approach, combining systemic and ocular assessments, is crucial to establish diagnostic clarity and refine therapeutic approaches, especially in the complex clinical scenarios often presented by inpatients with systemic infections. Ultimately, adopting this strategic framework aims to promote better patient outcomes through informed and timely intervention strategies.

## Figures and Tables

**Table 1:** Systemic bacterial infections: principal etiological agents and most common ocular fundus findings.

Etiological Agent (Systemic Condition)	Ocular Fundus Findings	Onset of Ocular Findings
*Streptococcus* spp. and *Staphylococcus* spp. (sepsis or endocarditis)	Roth spots, hemorrhages, endophthalmitis, chorioretinitis	Early
*Neisseria meningitidis* (meningitis)	Papilledema, hemorrhages	Early
*Mycobacterium tuberculosis* (tuberculosis)	Chorioretinitis, choroid tubercles, retinal vasculitis, panuveitis	Late
*Nocardia* spp. (sepsis)	Chorioretinitis, subretinal abscesses	Early
*Treponema pallidum* (syphilis)	Chorioretinitis, optic neuritis placoid lesions, retinal necrosis, vasculitis, panuveitis	Late
*Bartonella* spp. (bartonellosis)	Chorioretinitis, optic neuritis, focal retinitis, serous retinal detachment, vitritis	Late

**Table 2:** Systemic viral infections: principal etiological agents and most common ocular fundus findings.

Etiological Agent	Ocular Fundus Findings	Onset of Ocular Findings
Cytomegalovirus (CMV)	CMV retinitis, hemorrhages	Early in severe immunosuppresion
Herpes Simplex Virus (HSV) and Varicella-Zoster Virus (VZV)	Acute retinal necrosis (ARN), progressive outer retinal necrosis (PORN), retinitis, choroiditis	Early
Human Immunodeficiency Virus (HIV) with no other associated infections	Cotton-wool spots, microangiopathy	Late
SARS-CoV-2 (COVID-19)	Still no evidence of specific findings	Reported Early or Late

**Table 3:** Systemic fungal and parasitic infections: principal etiological agents and most common ocular fundus findings.

Etiological Agent	Ocular Fundus Findings	Onset of Ocular Findings
*Candida* spp. (candidiasis)	Retinitis, vitritis, endophthalmitis	Early/Late
*Toxoplasma gondii* (toxoplasmosis)	Retinochoroiditis (acutely or through reactivation); grey-white retinal necrosis with adjacent choroiditis and vitritis	Early Late (reactivation)
*Toxocara canis*/cati (toxocariasis)	Retinal granuloma, epiretinal membrane formation, macular edema, vitritis	Early/Late
*Plasmodium* spp. (malaria)	Retinal whitening, orange or white discoloration of vessels, hemorrhages, and potentially papilledema	Early

## Data Availability

Not applicable.
